# Water Intake in a Sample of Greek Adults Evaluated with the Water Balance Questionnaire (WBQ) and a Seven-Day Diary

**DOI:** 10.3390/nu8090559

**Published:** 2016-09-10

**Authors:** Adelais Athanasatou, Olga Malisova, Aikaterini Kandyliari, Maria Kapsokefalou

**Affiliations:** Unit of Human Nutrition, Department of Food Science and Human Nutrition, Agricultural University of Athens, 75 Iera Odos Str., Athens 11855, Greece; dathanasatou@gmail.com (A.A.); olgamalisova@yahoo.gr (O.M.); katerinakand@hotmail.com (A.K.)

**Keywords:** total water intake, beverages consumption, seven-day diary record, water balance questionnaire

## Abstract

Awareness on the importance of hydration in health has created an unequivocal need to enrich knowledge on water intake of the general population and on the contribution of beverages to total water intake. We evaluated in the past water intake in a sample of Greek adults using two approaches. In study A, volunteers completed the Water Balance Questionnaire (WBQ), a food frequency questionnaire, designed to evaluate water intake (*n* = 1092; 48.1% males; 43 ± 18 years). In study B, a different population of volunteers recorded water, beverage, and food intake in seven-day diaries (*n* = 178; 51.1% males; 37 ± 12 years). Herein, data were reanalyzed with the objective to reveal the contribution of beverages in total water intake with these different methodologies. Beverage recording was grouped in the following categories: Hot beverages; milk; fruit and vegetable juices; caloric soft drinks; diet soft drinks; alcoholic drinks; other beverages; and water. Total water intake and water intake from beverages was 3254 (SE 43) mL/day and 2551 (SE 39) mL/day in study A; and 2349 (SE 59) mL/day and 1832 (SE 56) mL/day in study B. In both studies water had the highest contribution to total water intake, approximately 50% of total water intake, followed by hot beverages (10% of total water intake) and milk (5% of total water intake). These two approaches contribute information on water intake in Greece and highlight the contribution of different beverages; moreover, they point out differences in results obtained from different methodologies attributed to limitations in their use.

## 1. Introduction

Reports that linked hydration with the maintenance of normal physical and cognitive functions [1] engendered the need for data on hydration of the general population and for public health advice on water intake.

Hydration reflects balance between water intake and loss. Water intake consists of water from a variety of sources; namely, drinking water, beverages, and fluid and solid foods. In most studies, drinking water and beverages contribute approximately 80%, and solid and fluid foods approximately 20% to water intake [2,3,4]. Water loss consists mainly from excretion of water in urine, respiratory water, feces, and sweat [2].

New studies focus on evaluating water intake in the general population in different countries using either new data or retrospective analyses of older studies, thus building information on water intake worldwide [4,5,6,7,8]. For example, total water intake is 1307 mL/day in adults aged 20–54 years, and 1198 mL/day in senior adults in France [6,9], and 3563 mL/day in the USA [10]. Overall, these differences are expected to some extent because water intake reflects environmental conditions and physical activity levels [11] that vary in different countries or population groups. Moreover, water intake is influenced by diverse dietary habits, the availability of a variety of beverages in local markets, and the adoption of drinking-friendly policies in public and private spaces (schools, working environments, hospitals, etc.). In this context, the recording of information on the contribution of different beverages in total water intake deserves attention. For example, in many countries drinking water is the most popular beverage [9,10] but in others, such as in the UK, hot beverages [5] are preferable.

Apart from differences amongst countries, the methodological tools used for evaluating water intake in these studies are inconsistent, some use seven-day diaries, 24 h recalls, or food frequency questionnaires [12]. Moreover, most of these studies used tools that were not specifically designed to evaluate water intake. Therefore, they may not fully capture the consumption of water or of other beverages [13].

Limited information on fluid intake is available for the Greek population [3,8,14]. Cohort studies conducted in Greece do not include the evaluation of water intake [15,16,17]. We have conducted two studies in the past with the objective to evaluate water intake in a sample of the Greek population. In these studies we analyzed total water intake without specific reference to the variety of beverages consumed. There is a need to obtain this important information because a higher variety has been linked to a higher total water intake [5]. Moreover, these studies used different methodologies; the study of Malisova et al. [8] used the WBQ [18], a semi-quantified food frequency questionnaire and the study of Malisova et al. [3] used a 7-day diary record. There is a need to carefully observe and comment on the use of different tools in the evaluation of water intake.

The objectives of the present study were to reanalyze the existing databases in order: (a) to report water intake and the type of beverages consumed in a sample of Greek adults using a food frequency questionnaire and a seven-day diary; and (b) to compare the water intake recorded from these two approaches, i.e., a semi-quantified food and fluids frequency questionnaire or a seven-day diary record.

## 2. Materials and Methods

We reanalyzed data from two existing databases; data were obtained from a semi-quantified food frequency questionnaire (study A), and from seven-day diaries (study B). The sample of studies A and B is composed of different subjects. A total of 1270 subjects from the metropolitan area of Athens (Greece) were included in these analyses.

In Study A [8] we used the Water Balance Questionnaire (WBQ) [18], a self-administrated semi-quantified food frequency questionnaire specially designed and validated with urine hydration biomarkers and three-day diaries to estimate water intake from all sources. The exclusion criteria were disease in relation to water balance, including urinary tract infection, kidney disease, and diabetes. All volunteers were informed on the objectives of the study and the procedures involved, and signed an informed consent. In 1092 healthy subjects 18–75 years (age: 43 ± 18 years; males 48.1%) water intake was estimated from the consumption of fifty-eight foods and from drinking water or beverages recorded in detail as glasses, bottles, or cups consumed per day. Employment status, education level, and levels of physical activity estimated from the International Physical Activity Questionnaire (IPAQ) questionnaire [19] were also considered. Height and weight of subjects was measured. Water from solid and fluid foods, recorded from the WBQ, was calculated using data from the USDA National Nutrient Database by multiplying the content in water given from the USDA of any food or beverage (in g or mL) with the portion size (in g or mL) and the number of times that the portion was consumed in the last month.

In study B [3] we used a seven-day diary to record detailed information on foods, and beverages intake. The study protocol had a 24 h urine collection for the same seven days in order to assess hydration status. Exclusion criteria were disease (diabetes insipidus, renal, liver, gastrointestinal, cardiac, pulmonary, or muscle-skeletal diseases), pregnancy, lactation, hypertension, taking diuretic drugs, and following a high-protein or hypocaloric diet. Written informed consent was obtained from all subjects. The recruitment strategy included invitations sent by email to the non-academic and academic personnel; uploaded on social media and published in local newspapers; uploaded on internet sites related to nutrition; and sent by email to other academic and social work institutions. A total of 178 healthy subjects 18–65 years (age: 37 ± 12 years; males 51.1%) recorded the type and amount of food and/or fluid consumption, time, and place immediately after it happened in order to avoid misreporting. Employment status, education level, tobacco use, and levels of physical activity estimated from the International Physical Activity Questionnaire (IPAQ) questionnaire [19] were also considered. Height and weight of subjects was measured. Water from solid and fluid foods, recorded from the seven-day diaries was calculated using the data from the Diet Analysis plus version 6.1 software (ESHA Research, Wadsworth Publishing Co. Inc., Salem, OR, USA).

### 2.1. Data Analysis

(a) Subjects from studies A and B with anomalous values of energy intake [20] were excluded from the analyses; below 500 calories for females and 800 calories for males, above 3500 calories for females and 4000 calories for males.

(b) Beverage consumption was combined in the eight following categories: (1) hot beverages (including tea and coffee); (2) milk (including regular, light, and chocolate milk); (3) fruit and vegetable juices (including nectar, fresh, and mix juices); (4) caloric soft drinks; (5) diet soft drinks; (6) alcoholic drinks; (7) water (including tap and bottled); and (8) other beverages (i.e., non-alcoholic beer). Total water intake was calculated from the moisture content in foods and the total beverages intake. Beverage intake was the sum of the amounts of these eight categories.

(c) The variety score was calculated as the sum of the beverages consumed from the eight different categories with a minimum value of “0” and a maximum value of “8”.

(d) Daily water intake of males and females was compared to the European Food Safety Authority (EFSA) Dietary Reference Values for Adequate Intake of water for males and females (2.5 L and 2.0 L, respectively) [2]. Nordic and German-speaking countries take the approach of inadequate water intake when it is less than 1 g per kilocalorie of energy requirement [2]. Therefore, we used three approaches to define adequate water intake in order to estimate the proportion of subjects that have a low total water intake. A classification based on the adequate intake value, defined by the EFSA, was considered as Criterion 1. Criterion 2 was considered as a ratio of total water intake and energy intake higher than 1. The combination of both (Criterion 1 and 2) was considered as the final criterion (Criterion 3).

### 2.2. Statistics

Continuous variables are expressed as the mean (standard error) for variables following normal distribution. Normal distribution of all continuous variables was tested with the Shapiro–Wilk parametric test or graphically assessed by histograms. All variables were found to be normal. Correlations were evaluated using Pearson’s correlation coefficient. Partial correlations between water intake, energy intake, and beverage consumption adjusted for age, gender, body weight, and physical activity were calculated by the use of a variety score. Differences between genders and age groups were observed using Student’s *t*-test. We deemed statistical significance at α = 0.05. Statistical analysis was performed by SPSS package, version 18 (SPSS Inc., Chicago, IL, USA).

## 3. Results

The age, gender distribution, and characteristics in details of subjects of study A are presented in Table 1.

The contribution of foods and beverages in detail to water intake (g/day) and energy intake (kcal/day) are presented in Table 2. Mean water intake was 3387 g/day (SE 46), while for males, was 3531 g/day (SE 71), and for females was 3253 g/day (SE 58). Beverage contribution to water intake was 78%, 80%, and 78% for the total sample, males, and females respectively. Mean energy intake was 1911 kcal/day (SE 26) for the total sample, 1975 kcal/day (SE 45) for males, and 1852 kcal/day (SE 31) for females, while the contribution of beverages to total energy intake was 24%, 26%, and 22% for the total sample, males, and females respectively. Finally, water was the most popular beverage consumed, followed by hot beverages, alcoholic drinks, and milk (Table 2).

Table 3 presents the contribution of foods and beverages in detail to water intake (g/day) and energy intake (kcal/day) by age groups for each gender. Differences were observed between two age groups for both genders for total water intake (*p* < 0.001), for water from beverages (*p* < 0.001) and for total beverages consumption (*p* < 0.001). Differences were also observed for beverage types, in particular for fruit and vegetable juices, for caloric soft drinks, for water, and for other non-alcoholic beverages.

Correlations between water intake, energy intake, and beverage consumption were highlighted in Table 4. Total water intake was strongly correlated with energy intake from beverages and total energy intake (*r* = 0.636, *r* = 0.618), while water intake from beverages was correlated moderately with energy intake from beverages (*r* = 0.589). Positive correlations were observed amongst total water intake and water intake from beverages (*r* = 0.914), and beverage weight (*r* = 0.957). Fruit and vegetable juice, alcoholic drinks, and water had a moderate correlation with total water (*r* = 0.389, 0.461 and 0.687, respectively). Additionally, caloric soft drinks and alcoholic drinks had a moderate correlation with total energy intake (*r* = 0.309, *r* = 0.516). The variety score was positively correlated with total water intake and water intake from beverages (*p* < 0.001 in both cases).

Table 5 presents detailed characteristics for the population of study B.

The contribution of foods and beverages to water (g/day) and energy intake (kcal/day) is presented in Table 6 for males, females, and totally. Mean daily water intake was 2349 g (SE 59), while for males it was 2517 g (SE 91), and for females it was 2174 g (SE 71). Beverages were the main contributors to water intake (79% for males, and 76% for females). The contribution of all types of beverages to water intake was similar for males and females (*p* values are provided in Table 6). Mean energy intake was 1780 kcal/day (SE 36), while the contribution of foods to total energy intake was approximately 78% for the total sample.

Males had a higher total water intake and water intake from beverages (*p* = 0.003 for both) as presented in Table 6. Moreover, males consumed more beverages than females (1999 g against 1692 g), which reflect a higher consumption of water (tap or bottled) (*p* = 0.007). Water was by far the most popular beverage consumed for both genders, followed by hot beverages, milk, and alcoholic drinks.

In Table 7 are presented the intakes of water, energy and beverage type the first three days of the experiment, as well as during the seven-day period. Total water intake, water intake from beverages, and energy intake decreased in the extended period of the study. The seven-day period revealed a higher variety score for beverages consumed of 5 (SD 1) and a higher intake of alcoholic drinks.

Total water intake was strongly correlated with beverage weight (*r* = 0.953) and water from beverages (*r* = 0.952). The correlation of total water intake with water from foods was very weak (*r* = 0.29, Table 8). Total water intake from all sources was correlated weakly with total energy intake (*r* = 0.265), and water intake from beverages is also correlated weakly with energy intake from beverages (*r* = 0.230). Milk, fruit and vegetable juice, alcoholic drinks, and caloric soft drinks had a moderate correlation with total energy from beverages (*r* = 0.46, 0.43, 0.43, and 0.24, respectively). The variety score of beverages consumed in a day was positively correlated with total water intake (*r* = 0.169), water and energy intake from beverages (*r* = 0.214 and 0.316, respectively).

Finally, in the present study the classification of total water intake is presented in Table 9. Seventy-five percent of males from study A and 40% from study B followed the scientific opinion of EFSA for adequate daily water intake (2.5 L/day; Criterion 1). The adherence of females (2.0 L/day; Criterion 1) was 83% in the sample of study A and 62% in study B. Ninety-six percent of the subjects from study A and 80% of subjects from study B fulfilled Criterion 2 (1 g of water per 1 kcal of energy intake).

## 4. Discussion

The present study reports and comments on data for water intake from all sources (foods and beverages) using the WBQ, a semi-quantified food and fluid frequency questionnaire (study A) and a seven-day diary record (study B) in healthy Greek adults aged 18–75 years living in the metropolitan area of Athens.

The main finding of our study is that total water intake was 3254 (SE 43) g/day in study A and 2349 (SE 59) g/day in study B. This finding draws attention not only because it contributes data for water intake in Greece, but also because it reveals deviation in findings when using different research tools.

Total water intake in other countries ranges from 1488 mL/day in China to 3563 mL/day in the USA [4,5,6,7,9,10,21,22,23,24]. Deviation in water intake in different countries may reflect between country differences in dietary habits, lifestyle choices, and environmental conditions [25], but also between study differences in the choice of method used to evaluate water intake.

The research tools that are used in most studies are three- or seven-day diary records, or 24 h recall or food frequency questionnaires [24], but commonly these are designed to evaluate food intake and not water intake; studies in large population groups, such as the European Prospective Investigation into Cancer and Nutrition (EPIC) study and the National Health and Nutrition Examination Survey (NHANES) study, have been designed to assess macro and micronutrient intakes and not total water or beverages intake. Therefore, they may not fully capture water intake because the consumption of some beverages is underestimated by the individual or the interviewer [13]. For example, the food intake diaries or 24 h recalls record eating occasions around meals and snacks, but not all drinking occasions, while fluid-specific, records report two more drinking occasions per day not with meals or snacks. These are less likely to be included in a 24 h recall [26]. A growing number of studies having as their primary outcome the estimation of total water intake is now published [3,8,23,24,27]. Research tools that are designed specifically to record water intake exist [18,21,24]. It appears that these report a higher total fluid intake compared to tools that are not specifically designed to record water intake [21,24].

In data presented herein, this observation is confirmed. In study A the WBQ, which registers in detail all sources of fluid consumption, recorded a higher water intake by approximately 900 mL/day than in study B, although subjects completing the seven-day diaries were instructed to record all drinking occasions. The difference between study A and B may be attributed to a variety of factors: WBQ was administered to 1092 subjects while the seven-day diaries were administered to 178 subjects, although of the same distribution in terms of age, sex, season, and location; WBQ records water intake for beverages for the previous month, while seven-day diaries record water intake for one week; WBQ, designed specifically for recording fluid intake, embeds a food frequency questionnaire with 23 questions for beverage intake including all types of hot and cold beverages, alcoholic, and non-alcoholic beverages, that are usually consumed in Greece; questions in the WBQ were expressed as the number of glasses, while continuous data (mL per drinking occasion) were collected in seven-day diary records; the seven-day diary was part of an elaborate protocol including 24 h urine collection for all seven days. This systematic urine collection in study B may be intruding and may alter routine behavior, including drinking.

There is not yet a gold standard method to assess water intake. Contributing to the discussion on the appropriateness of research tools evaluating water intake, the exposure study of Mons, et al. [28] concluded that the best method to collect water intake data is a 3–4 days diary record or, if not feasible, two or more 24 h recalls are preferable to food frequency questionnaires. It must be noted that the WBQ was validated with three-day diary records [18]. Mons, et al. [28] also suggests that an extended period may result to less accurate reporting. In order to confirm this argument we retrospectively analyzed diary records from study B and compared the recordings of three and in seven days. We observed a decreased total water intake and water intake from beverages in seven days. Others [29,30,31] also supported that food frequency questionnaires report a higher water intake than the diary records.

International organizations define adequate water intake based on data collected in various population groups. In Europe, EFSA [2] defines adequate water intake from all sources at 2 L for females and 2.5 L per day for males. In the USA, based on daily dietary recruitment (DRI), Institute of Medicine (IOM) suggests adequate intake range 2.7–3.7 L per day in adults, with men to require 1 L more [32]. Adequate intake may be adjusted when water requirements are increased according to physical activity levels and environmental conditions [32,33,34]. It may be expected that new data collected using improved validated methodology specific for evaluating water intake in different population groups and in different countries may lead to resetting values for adequate water intake by different organizations.

The discrepancy of results obtained from different tools is clearly confusing, when it comes to observing compliance with EFSA adequate intakes. For example, 83% of females and 75% of males from study A, and 62% of females and 40% of males from study B, complied with the EFSA adequate water intake. In the study of Ferreira-Pego, et al. [24], averaging data from 13 countries, 40% of men and 60% of women complied with the EFSA adequate intakes for water intake from fluids. In the ANIBES study [23] performed in Spain, 21% of women and 12% of men complied with EFSA adequate intakes. In general, women exhibit a healthier pattern of eating and food choices than men [35]. Women seem to be more reflective about health issues and foods. Adults that adopt a healthier dietary pattern usually have a healthier fluid pattern (higher consumption of water and total fluids) [36].

Another important finding is that, despite differences in volumes when recording total water intake using the WBQ and seven-day diaries in study A and B, respectively, the contribution in water intake of foods and beverages, as well as of types of beverages, was similar in both studies. The importance of all sources, i.e., drinking water or beverages or moisture in solid foods in hydration [36] should be highlighted. This finding signifies that WBQ and seven-day diaries may evaluate the subjects’ choices in a similar manner. In particular, the contribution of beverages to total water intake was 78% in WBQ study and seven-day diaries study of foods was 22%, respectively. This finding accords with the scientific opinion of EFSA [2]. Similar findings in the contribution of foods and beverages to water intake were observed in the UK [5] and Indonesian [4] populations.

Beverages that were consumed in larger volumes in both studies A and B were water, by almost 10-fold, followed by hot beverages and milk. This finding is in accordance with the study of Armstrong, et al. [37] in which water consumed in similar volumes, as well as the study of Perrier, et al. [38] with water being the major contributor to fluid intake. These findings were not observed in the UK population, with hot beverages being the most popular beverage. The variety in beverage choice has been considered a factor linked to water intake. An important finding from our study is that the variety score, using both tools, is positively correlated with total water intake (*p* < 0.001, *p* = 0.005 respectively) and water intake from beverages (*p* < 0.001, *p* = 0.005 respectively).

The results of this study may be exploited in view of a number of limitations. The WBQ that was used in study A estimates the usual food intake over a month, but details of intake are not measured, such as the size of the portion consumed. In addition, retrospective methods, such as 24 h recall and food frequency questionnaires, depend on memory and recall ability of the applicant [27]. Both methodologies require updated and extended information for food composition data, which is limited in Greece. It should be noted that subjects of study B that completed the seven-day diaries had to follow a demanding protocol that required the collection of samples from all urination on a 24 h basis for seven consecutive days. It appears that approaches such as three- or seven-day diary records that require a collection of a large amount of data from the subject results in reduced compliance and may underestimate the fluid intake [26]. New research developments that introduce electronic recording of dietary intake [28] attempt to maximize the compliance of the subject; however, these are not yet used extensively in water intake.

## 5. Conclusions

In conclusion, water intake using the WBQ recorded a higher water intake than the seven-day diaries in a sample of Greek adults, yet both methodologies found that the beverages that were consumed in larger volumes were water, hot beverages, and milk. This work implies caution when interpreting data obtained from different approaches and highlights the need for concerted efforts towards developing a robust, validated methodology for the evaluation of water intake in the general population.

## Figures and Tables

**Table 1:** Descriptive characteristics of subjects that completed the WBQ (*n* = 1092).

			**Total**	**%**	**Male**	**%**	**Female**	**%**
		Total	1092	100	532	48.1	575	51.9
Age (years)	18–39	Count	488	44.7	230	44.1	258	45.3
	40–64	Count	390	35.7	187	35.8	203	35.6
	65–75	Count	214	19.6	105	20.1	109	19.1
Unemployed		Count	139	13.2	61	12.0	78	14.1
Level of physical activity	Inactive	Count	71	6.4	37	7.0	34	5.9
	Active	Count	1036	93.6	495	93.0	541	94.1
Level of education	Primary or less	Count	129	14.1	47	10.8	82	17.2
	Secondary	Count	240	26.3	131	30.1	109	22.8
	Tertiary or University	Count	544	59.6	257	59.1	287	60.0
Weight (kg)		Mean (SE)	72.1 (0.4)		81.2 (0.5)		63.6 (0.5)	
Height (cm)		Mean (SE)	169.5 (0.3)		176.2 (0.3)		163.1 (0.3)	
BMI (kg/m^2^)		Mean (SE)	25.07 (0.12)		26.22 (0.16)		23.98 (0.18)	
BMI class (kg/m^2^)	Underweight	Count	28	2.8	3	0.6	25	4.8
	Normal weight	Count	513	50.9	188	38.6	325	62.5
	Overweight	Count	351	34.9	218	44.8	133	25.6
	Obese	Count	115	11.4	78	16.0	37	7.1

**Table 2:** Contribution of food and beverages to total water and energy intake of subjects using the WBQ (*n* = 1092).

		Total Weight Consumed (g/Day) GRAMS				Contribution to Energy Intake (kcal/Day) KCAL				Contribution to Water Intake (g/Day) WATER			
		Total	Male	Female	*p*^1^	Total	Male	Female	*p*^2^	Total	Male	Female	*p*^3^
	Count	1107	532	575		1107	532	575		1107	532	575	
All food and drink	Mean (SE)	3387 (46)	3531 (71)	3253 (58)	<0.01	1911 (26)	1975 (45)	1852 (31)	0.02	3254 (43)	3404 (66)	3116 (55)	0.001
Food only	Mean (SE)	831 (14)	817 (22)	843 (18)	0.37	1453 (21)	1471 (35)	1437 (26)	0.43	706 (12)	683 (19)	727 (16)	0.07
Beverages only	Mean (SE)	2668 (40)	2826 (62)	2522 (50)	<0.01	460 (12)	508 (20)	416 (12)	<0.001	2551 (39)	2725 (61)	2390 (50)	<0.001
Hot beverages	Mean (SE)	283 (9)	261 (12)	304 (12)	0.12	149 (4)	141 (7)	157 (6)	0.08	330 (9)	307 (13)	351 (13)	0.02
Milk	Mean (SE)	176 (6)	175 (10)	178 (8)	0.84	115 (5)	113 (6)	116 (6)	0.66	160 (6)	158 (9)	162 (7)	0.69
Fruit and Vegetable Juices	Mean (SE)	128 (8)	138 (12)	119 (11)	0.23	65 (4)	69 (6)	61 (5)	0.34	119 (7)	126 (11)	112 (9)	0.34
Caloric soft drink	Mean (SE)	71 (5)	89 (8)	54 (5)	<0.001	27 (2)	34 (3)	21 (2)	<0.001	64 (4)	80 (7)	48 (4)	<0.001
Diet soft drink	Mean (SE)	58 (4)	57 (6)	58 (6)	0.86	5 (0)	7 (1)	4 (0.4)	<0.001	52 (4)	47 (5)	57 (7)	0.25
Alcohol	Mean (SE)	267 (17)	310 (29)	227 (18)	0.14	95 (8)	140 (15)	54 (5)	<0.001	146 (13)	215 (26)	82 (8)	<0.001
Water	Mean (SE)	1671 (30)	1779 (43)	1571 (40)	<0.001	-	-	-	<0.001	1671 (30)	1779 (43)	1571 (40)	<0.001
Other non-alcoholic beverages	Mean (SE)	15 (2)	20 (3)	10 (1)	<0.01	3 (0)	5 (1)	54 (5)	<0.01	9 (1)	14 (2)	5 (1)	<0.001

*p*-values derived through Student’s *t*-test between genders.

**Table 3:** Total water intake and beverage consumption (g/day) by age group of subjects using the WBQ (*n* = 1092).

	Male				Female			
	Age Group (Years)				Age Group (Years)			
		18–64	65–75	*p*^1^		18–64	65–75	*p*^2^
	(A)	(B)	(C)		(D)	(E)	(F)	
	532	419	103		575	466	104	
Total Water intake from food and beverages mean (SE)	3404 (66)	3568 (75)	3030 (104)	<0.001	3116 (55)	3243 (62)	2658 (110)	<0.001
Water from food mean (SE)	683 (19)	683 (22)	738 (33)	0.24	727 (16)	738 (17)	693 (42)	0.27
Water from beverages mean (SE)	2725 (61)	2886 (69)	2315 (100)	<0.001	2390 (50)	2505 (57)	1997 (83)	<0.001
Total beverages consumption mean (g/day) (SE)	2826 (62)	3012 (70)	2323 (97)	<0.001	2522 (50)	2656 (56)	1997 (84)	<0.001
of which (g/day)								
Hot beverages mean (g/day) (SE)	307 (13)	265 (13)	269 (30)	0.89	351 (13)	317 (14)	260 (26)	0.08
Milk mean (g/day) (SE)	158 (9)	179 (11)	178 (19)	0.98	162 (7)	180 (9)	174 (20)	0.76
Fruit and Vegetable Juices mean (g/day) (SE)	126 (11)	158 (14)	72 (12)	<0.001	112 (9)	131 (13)	74 (15)	0.04
Caloric soft drink mean (g/day) (SE)	80 (7)	101 (10)	49 (8)	<0.001	48 (4)	61 (6)	26 (7)	<0.001
Diet soft drink mean (g/day) (SE)	47 (5)	56 (6)	*65 (17)*	0.62	57 (4)	61 (6)	43 (16)	0.21
Alcohol mean (g/day) (SE)	215 (26)	329 (34)	264 (58)	0.34	82 (8)	255 (21)	91 (21)	<0.001
Water mean (g/day) (SE)	1779 (43)	1900 (50)	1442 (61)	<0.001	1571 (40)	1640 (47)	1323 (62)	<0.01
Other non-alcoholic beverages mean (g/day) (SE)	14 (2)	25 (4)	1 (1)	<0.001	5 (1)	11 (6)	2 (2)	0.07

*p*-values derived through Student’s *t*-test age groups.

**Table 4:** Partial correlations between water intake, energy intake and beverage consumption adjusted for age, gender, body weight, and physical activity using the WBQ (*n* = 1092).

	Total Water (from Food and Beverages) (g/Day)	Total Water from Beverages (g/Day)	Total Water from Food (g/Day)	Total Food Weight (g/Day)	Total Beverages Weight (g/Day)	Total Energy (kcal/Day)	Total Energy from Food (kcal/Day)	Total Energy (kcal/Day) from Beverages
Total water (g/day) (from food and beverages)	1	0.914 **	0.371 **	0.461 **	0.957 **	0.618 **	0.354 **	0.636 **
Total water (g/day) from beverages	0.914 **	1	0.117 **	0.167 **	0.958 **	0.493 **	0.089 **	0.589 **
Total water (g/day) from food	0.371 **	0.117 **	1	0.808 **	0.146 **	0.337 **	0.730 **	0.143 **
Total food weight (g/day)	0.461 **	0.167 **	0.808 **	1	0.184 **	0.370 **	0.781 **	0.238 **
Total beverages weight (g/day)	0.957 **	0.958 **	0.146 **	0.184 **	1	0.563 **	0.136 **	0.627 **
Total energy (kcal)	0.618 **	0.493 **	0.337 **	0.370 **	0.563 **	1	0.438 **	0.523 **
Total energy (kcal) from food	0.354 **	0.089 **	0.730 **	0.781 **	0.136 **	0.438 **	1	0.189 **
Total energy (kcal) from beverages	0.636 **	0.589 **	0.143 **	0.238 **	0.627 **	0.523 **	0.189 **	1
(1) Hot beverages (g/day)	0.297 **	0.222 **	0.165 **	0.235 **	0.252 **	0.144 **	0.153 **	0.395 **
(2) Milk (g/day)	0.147 **	0.111 **	0.165 **	0.172 **	0.107 **	0.104 **	0.156 **	0.229 **
(3) Fruit and vegetable juice (g/day)	0.389 **	0.379 **	0.082*	0.097 **	0.400 **	0.309 **	0.097 **	0.568 **
(4) Caloric soft drink (g/day)	0.224 **	0.230 **	−0.038	0.028	0.239 **	0.216 **	0.038	0.355 **
(5) Diet soft drink (g/day)	0.162 **	0.086 *	−0.002	0.069*	0.157 **	0.152 **	0.011	0.118 **
(6) Alcoholic drinks (g/day)	0.461 **	0.347 **	0.077 *	0.065	0.490 **	0.516 **	0.117 **	0.658 **
(7) Water (g/day)	0.687 **	0.804 **	0.049	0.05	0.744 **	0.219 **	−0.005	0.034
(8) Other non-alcoholic beverages (g/day)	0.191 **	0.083 *	0.017	0.146 **	0.164 **	0.135 **	0.064	0.110 **
Variety score	0.160 **	0.170 **	0.007	0.112 **	0.190 **	0.136 **	0.096 **	0.194 **

** Correlation is significant at the 0.01 level; * Correlation is significant at the 0.05 level.

**Table 5:** Descriptive characteristics of subjects that completed seven-day diaries (*n* = 178).

			Total	%	Male	%	Female	%
		Total	178	100	91	51.1	87	48.9
Age group (years)	18–39	Count	103	57.9	56	61.5	47	54
	40–64	Count	75	42.1	35	38.5	40	46
Unemployed		Count	139	13.1	61	12	78	14.1
Level of physical activity	Inactive	Count	109	61.2	58	63.4	51	59.0
	Active	Count	69	38.8	33	36.6	36	41.0
Level of education	Primary or less	Count	1	1	0	0	1	2.7
	Secondary	Count	2	2	1	1.6	1	2.7
	Tertiary or University	Count	95	96.9	60	98.4	35	94.6
Tobacco	Yes	Count	41	23	20	22	21	24.1
	No	Count	137	77	71	78	66	75.9
Weight (kg)		Mean (SE)	75.53 (3.38)		82.89 (6.62)		68.44 (1.57)	
Height (cm)		Mean (SE)	1.70 (0.01)		1.73 (0.01)		1.67 (0.01)	
BMI Class (kg/m^2^)	Normal weight	Count	104	58.4	50	55.4	53	61.3
	Overweight	Count	48	26.8	26	28.4	22	25.3
	Obese	Count	26	14.8	15	16.2	12	13.3

**Table 6:** Contribution of foods and beverages to water and energy intake using seven-day diaries (*n* = 178).

		Contribution to Water Intake (g/Day)			Contribution to Energy Intake (kcal/Day)				
		Total	Male	Female	*p*^1^	Total	Male	Female	*p*^2^
	Count	178	91	87		178	91	87	
All food and drink	Mean (SE)	2349 (59)	2517 (91)	2174 (71)	0.003	1780 (36)	1890 (51)	1667 (46)	0.002
Food only	Mean (SE)	504 (17)	501 (21)	508 (27)	0.848	1551 (31)	1594 (47)	1425 (37)	0.005
Beverages only	Mean (SE)	1826 (57)	1990 (90)	1653 (63)	0.003	206 (9)	216 (12)	194 (12)	0.199
Hot beverages	Mean (SE)	286 (17)	282 (22)	291 (26)	0.779	127 (8)	125 (10)	129 (12)	0.779
Milk	Mean (SE)	119 (8)	116 (12)	122 (12)	0.721	117 (8)	114 (12)	120 (11)	0.721
Fruit and vegetable juice	Mean (SE)	63 (6)	57 (8)	69 (8)	0.272	36 (3)	32 (5)	39 (4)	0.272
Caloric soft drink	Mean (SE)	27 (4)	30 (5)	24 (6)	0.486	12 (2)	13 (2)	11 (3)	0.486
Diet soft drink	Mean (SE)	23 (6)	33 (10)	12 (5)	0.075	1 (0)	1 (0)	0 (0)	0.075
Alcoholic drinks	Mean (SE)	81 (9)	84 (12)	77 (12)	0.696	142 (15)	147 (21)	136 (22)	0.696
Water	Mean (SE)	1170 (54)	1310 (86)	1023 (61)	0.007	- -	- -	- -	-
Other beverages	Mean (SE)	18 (3)	23 (6)	12 (2)	0.096	5 (1)	6 (2)	3 (1)	0.096

*p*-values derived through Student’s *t*-test between genders.

**Table 7:** Water and energy intake of subjects the first three days and the seven days of the experiment using day diaries (*n* = 178).

Variable	3 Days	7 Days	*p*
Total water intake (mL/day)	2412 (63)	2351 (59)	0.005
Water intake from beverages (mL/day)	1869 (60)	1826 (57)	0.027
Water intake from foods (mL/day)	535 (19)	505 (17)	0.009
Total energy intake (kcal/day)	1818 (38)	1775 (35)	0.017
Energy intake from beverages (kcal/day)	201 (9)	207 (9)	NS
Energy intake from foods (kcal/day)	1573 (36)	1512 (31)	0.011
Hot beverages (mL/day)	302 (19)	290 (17)	NS
Milk (mL/day)	138 (11)	143 (10)	NS
Fruit and vegetable juice (mL/day)	79 (8)	72 (8)	NS
Caloric soft drinks (mL/day)	29 (6)	31 (4)	MS
Diet soft drinks (mL/day)	28 (8)	26 (7)	NS
Alcoholic drinks (mL/day)	85 (10)	100 (11)	0.036
Water (mL/day)	1233 (55)	1176 (54)	0.004
Other beverages (mL/day)	16 (3)	20 (4)	0.159
Variety score	4	5	0.0001

*p*-values derived through Student’s *t*-test between three and seven days of the experiment.

**Table 8:** Partial correlations between water intake, energy intake, and beverage consumption adjusted for age, gender, body weight, and activity from seven-day diaries (*n* = 178).

	Total Water Intake (g/Day)	Total Water from Beverages (g/Day)	Total Water from Food (g/Day)	Total Beverages Weight (g/Day)	Total Energy (kcal/Day)	Total Energy from Beverages (kcal/Day)	Total Energy from Food (kcal/Day)
Total water (from food and beverages) (g/day)	1	0.952 **	0.291 **	0.953 **	0.265 **	0.213 *	0.222 **
Water from beverages (g/day)	0.952 **	1	0.006	0.959 **	0.142	0.230 **	0.094
Water from food (g/day)	0.291 **	0.006	1	0.142	0.407 **	0.013	0.446 **
Beverage weight (g/day)	0.953 **	0.959 **	0.142	1	0.189 *	0.291 **	0.140
Total energy (kcal)	0.265 **	0.142	0.407 **	0.189 *	1	0.394 **	0.885 **
Energy from beverages (kcal)	0.213 *	0.230 **	0.013	0.291 **	0.394 **	1	0.146
Energy from food (kcal)	0.222 **	0.094	0.446 **	0.140	0.885 **	0.146	1
(1) Hot beverages (g/day)	0.092	0.116	−0.048	0.131	−0.078	−0.101	−0.020
(2) Milk (g/day)	0.080	0.010	0.211 *	0.100	0.199 *	0.460 **	0.089
(3) Fruit and vegetable juice (g/day)	0.273 **	0.286 **	−0.030	0.314 **	0.277 **	0.428 **	0.145
(4) Caloric soft drink (g/day)	0.147	0.098	0.219 **	0.206 *	0.239 **	0.376 **	0.167 *
(5) Diet soft drink (g/day)	0.140	0.145	−0.006	0.145	−0.002	−0.005	0.066
(6) Alcoholic drinks (g/day)	0.254 **	0.279 **	−0.005	0.276 **	0.125	0.433 **	0.039
(7) Water (g/day)	0.898 **	0.909 **	0.115	0.919 **	0.110	0.085	0.094
(8) Other beverages (g/day)	0.251 **	0.245 **	0.077	0.265 **	0.161	0.264 **	0.074
Variety score	0.167 *	0.214 *	−0.098	0.250 **	0.121	0.316 **	0.053

** Correlation is significant at the 0.01 level; * Correlation is significant at the 0.05 level.

**Table 9:** Combined classification for the total water intake (TWI) following established criteria.

Classification of Total Water Intake	Study	Males	Females
CRITERION 1 (%)	A	75	83
	B	40	62
CRITERION 2 (%)	A	97	96
	B	79	81
CRITERION 3 (1 and 2) (%)	A	74	80
	B	40	60

(1) Criterion 1: TWI > 2.5 L males, > 2 L females (aged 14 to 75 years); (2) Criterion 2: Ratio of total water/total energy intakes > 1; (3) Criterion 3: Both criteria.
